# Family medicine as an emerging specialty: a Pakistani perspective and the RCGP International Overseas Network

**DOI:** 10.3399/bjgpopen18X101601

**Published:** 2018-08-08

**Authors:** Faridah Amin, Saniya R Sabzwari

**Affiliations:** 1 Assistant Professor and Head of Family Medicine Department, Liaquat National Hospital, Karachi, Pakistan; 2 Associate Professor, Department of Family Medicine, Aga Khan University Hospital, Karachi, Pakistan

**Keywords:** ION, General Practice, Pakistan

From the heyday of the family doctor doing home visits to rapid rise of specialists caring for the masses, family medicine is making a comeback. This revival is not only in the western hemisphere but also in the East, specifically in developing countries.^1^ Here we hope to describe the journey of family medicine in Pakistan, and recent developments. The updates include change in policy, and in programme development both at academic and health provision levels.

Historically, Pakistan’s health sector has had both public and private arms with self-funding patients. The government subsidises health care, however quality of care is compromised because of problems in infrastructure, personnel, and policy, resulting in substandard care. A structure for free or cost-effective primary care leading to need-based secondary or tertiary care referral is not present, which is essential for an integrated, horizontal, and coordinated healthcare system. Another compounding factor in healthcare provision has been a strong focus on specialist care linked to secondary and tertiary healthcare systems, with insufficient development of primary care both in public and private sectors. All of this, coupled with low healthcare budget allotment, has resulted in ineffective healthcare systems, especially for the underprivileged population.^2^

The 18th amendment in the constitution of Pakistan in 2010 led to the handing over of responsibility for health care from the federal (central) to the provincial (regional) level. This devolution presented an opportunity for health sector reform at a more local level, catering to health needs of local communities. Though in its infancy, some success stories have emerged. The early realisation of the enormity of the task led to development of several public-private partnerships across the country, spread across administrative, resource, and policy levels.^3^ For example, recently a family medicine pilot project was launched through a public-private partnership between the provincial government and World Health Organization; a testimony to the acceptance and adoption of family medicine. In this project, a trained family physician serves as a core member of a multidisciplinary team to impart effective, quality primary care to the communities. Future linkage to a health insurance scheme offered by the provincial government is also anticipated.^4^

Another recent development by the medical licensing organisation of Pakistan (Pakistan Medical and Dental Council) is the recognition of family medicine as a primary care specialty in Pakistan, as in other developing countries of the world.^5^ Adoption of this plan was initially sluggish but has recently gained momentum. Postgraduate training programmes leading to qualifications like FCPS or MCPS (Fellow or Member of College of Physicians and Surgeons of Pakistan), and MRCGP International (International Member of the Royal College of General Practitioners) are gaining popularity, with rising number of candidates applying and obtaining certification after training.^6^

As health care is evolving and steps are being taken to strengthen primary care in developing countries, the dearth of trained primary care physicians remains a challenge.^7^ For example, in Brazil — where evidence has emerged regarding the 'family health strategy' as an effective primary care approach — a paucity of trained family physicians has limited the utility and extension of these services. To address these challenges, strategies are being made to introduce and expose undergraduate students to professional principles of family practice.^8^

Similarly, in Pakistan, another important development heralding a move towards strengthening family medicine was mandating family medicine as a required specialty in undergraduate medicine in 2014.^9^ Since then, a gradual introduction of clinical rotations in family medicine has been seen in medical colleges both from the public and private sector. The hope is that this landmark development (occurring almost 20 years after the first undergraduate family medicine curriculum was developed for a private medical university) will be one of the ways of addressing the shortage of trained faculty and physicians in family medicine.

The authors envision that over the years, as these changes evolve, a merging of these resources will occur, such as increasing physician resource to expand primary care services via public-private partnerships, providing enhanced services, building capacity for primary care by training physicians, and improving infrastructure and healthcare delivery. An example would be developing primary care programmes for high burden diseases like diabetes and hypertension in which academic institutions with primary care expertise plan, implement, and provide human resource, and the provincial government provides logistic and financial support. One hopes that such targeted programmes offered at a primary care level via existing basic health units and district level healthcare facilities would enhance efficient healthcare provision in communities, consequently reducing unnecessary load on tertiary health care and would allow delivery of comprehensive, accessible, and cost-effective health care.
